# Mobile Safety Alarms Based on GPS Technology in the Care of Older Adults: Systematic Review of Evidence Based on a General Evidence Framework for Digital Health Technologies

**DOI:** 10.2196/27267

**Published:** 2021-10-11

**Authors:** Maria Ehn, Matt X Richardson, Sara Landerdahl Stridsberg, Ken Redekop, Sarah Wamala-Andersson

**Affiliations:** 1 School of Innovation, Design and Engineering Mälardalen University Västerås Sweden; 2 School of Health, Care and Social Welfare Mälardalen University Eskilstuna Sweden; 3 University Library Mälardalen University Västerås Sweden; 4 Erasmus School of Health Policy and Management Erasmus Universiteit Rotterdam Rotterdam Netherlands

**Keywords:** GPS, older adults, dementia, evidence, digital health, NICE, mobile phone

## Abstract

**Background:**

GPS alarms aim to support users in independent activities. Previous systematic reviews have reported a lack of clear evidence of the effectiveness of GPS alarms for the health and welfare of users and their families and for social care provision. As GPS devices are currently being implemented in social care, it is important to investigate whether the evidence of their clinical effectiveness remains insufficient. Standardized evidence frameworks have been developed to ensure that new technologies are clinically effective and offer economic value. The frameworks for analyzing existing evidence of the clinical effectiveness of GPS devices can be used to identify the risks associated with their implementation and demonstrate key aspects of successful piloting or implementation.

**Objective:**

The principal aim of this study is to provide an up-to-date systematic review of evidence based on existing studies of the effects of GPS alarms on health, welfare, and social provision in the care of older adults compared with non–GPS-based standard care. In addition, the study findings were assessed by using the evidence standards framework for digital health technologies (DHTs) established by the National Institute for Health and Care Excellence (NICE) in the United Kingdom.

**Methods:**

This review was conducted according to the PRISMA (Preferred Reporting Items for Systematic Reviews and Meta-Analyses) guidelines. Primary studies published in peer-reviewed journals and gray literature from January 2005 to August 2020 were identified through searches in 13 databases and several sources of gray literature. Included studies had individuals (aged ≥50 years) who were receiving social care for older adults or for persons with dementia; used GPS devices as an intervention; were performed in Canada, the United States, European Union, Singapore, Australia, New Zealand, Hong Kong, South Korea, or Japan; and addressed quantitative outcomes related to health, welfare, and social care. The study findings were analyzed by using the NICE framework requirements for *active monitoring* DHTs.

**Results:**

Of the screened records, 1.6% (16/986) were included. Following the standards of the NICE framework, practice evidence was identified for the tier 1 categories *Relevance to current pathways in health/social care system* and *Acceptability with users*, and minimum evidence was identified for the tier 1 category *Credibility with health, social care professionals*. However, several evidence categories for tiers 1 and 2 could not be assessed, and no clear evidence demonstrating effectiveness could be identified. Thus, the evidence required for using DHTs to track patient location according to the NICE framework was insufficient.

**Conclusions:**

Evidence of the beneficial effects of GPS alarms on the health and welfare of older adults and social care provision remains insufficient. This review illustrated the application of the NICE framework in analyses of evidence, demonstrated successful piloting and acceptability with users of GPS devices, and identified implications for future research.

## Introduction

### Background

Health and welfare technologies (HWTs) are “technology-based interventions that aim at maintaining or promoting health, well-being, quality of life (QoL) and/or increasing efficiency in the service delivery system of welfare, social and health care services, while improving working conditions of the staff” [1]. This definition can be regarded as an integration of the Scandinavian term *welfare technology* [2] and the global concept *digital health* [3], including a broader definition of health according to the World Health Organization: “a state of complete physical, mental and social well-being and not merely the absence of disease or infirmity.” GPS alarms are examples of HWTs that aim to support users in independent activities of daily living, both indoors and outdoors, something that is needed by large groups of older adults who want to maintain independent living in spite of cognitive impairments, for example, persons with dementia in the early or middle stage. Globally, approximately 50 million people have dementia, and nearly 10 million new persons develop dementia every year [4]. The global population of persons with dementia is estimated to increase to 82 million in 2030 and to 152 million in 2050 [4].

GPS solutions enable the user to send an alarm to home care, family members, or both, either at home or outdoors. The user’s position can be localized through GPS coordinates when an alarm is sent. GPS alarms may also include a geofencing function that automatically generates an alarm if the user leaves a predefined geographical area. A European overview has identified ongoing implementation of GPS alarms in the municipal care of older adults in Sweden and Norway [5].

Research on the implementation of welfare technology in Swedish municipalities has identified several barriers to its use, including lack of supporting evidence of the benefits and positive outcomes [6]. Hence, evidence of positive outcomes of GPS alarms can justify large-scale implementation and confidence in their use.

When making implementation decisions, it is important to consider what kind of evidence is needed to make an informed decision. To date, there is no national Swedish evidence-based framework to support decision-making processes related to HWTs [7]. However, the National Institute for Health and Care Excellence (NICE) in the United Kingdom, together with relevant stakeholders, has produced an evidence standards framework for digital health technologies (DHTs) to ensure that new technologies can demonstrate clinically effective and economic value in a structured and transparent manner [8]. The framework classifies DHTs by function and thereby allows them to be stratified into evidence tiers based on the potential risk to users. For example, DHTs that track patient location can be classified as *active monitoring* and thereby stratified into evidence tier 3b in the framework. Good levels of evidence for tier 3 DHTs include evidence of credibility with health and social care professionals, relevance to current care pathways in health care systems, acceptability with users, equalities considerations in provision and use, accurate and reliable measurements and transmission of data, reliable information content, ongoing and planned continuous data collection to follow up on the use and value of the DHT, quality and safeguarding, and demonstration of its effectiveness according to intended outcomes. The framework has been applied in examples of case studies that demonstrate evidence of effectiveness and economic value of a number of DHTs [9]. These studies are based largely on information provided by the developers that has not been independently verified. Information from studies published in peer-reviewed journals and in gray literature can therefore strengthen the validity of evidence of DHTs.

Two previous scoping reviews of tracking technology in the care of older adults conducted by Røhne et al [10] and Neubauer et al [11] identified limited evidence in peer-reviewed scientific studies for the effects of GPS use on health, quality, and cost-efficiency among users and spouses and in the health and welfare sector [10] as well as pointed out that further research was needed to identify technologies with high levels of evidence for effectiveness and usability [11]. Moreover, a synthesizing review of empirical evidence on the broader use of GPS technologies by home-dwelling persons with dementia and their family caregivers (CGs), conducted by Bartlett et al [12], found only nontrial evidence and demonstrated the lack of large-scale studies. As GPS devices are currently being implemented in social care, it is important to investigate whether the evidence of their clinical effectiveness remains insufficient. By analyzing existing evidence of GPS devices using an established framework, the risks associated with their implementation and conclusions from successful piloting or implementations can be identified.

### Objectives

The aims of this review are to (1) systematically update evidence from existing studies of the effects of GPS alarms on the health, welfare, and social provision in receivers of care for older adults compared with non–GPS-based standard care and (2) review the findings using an established evidence framework for DHTs. Studies from both peer-reviewed journals and gray literature were included, and the findings were compared with the NICE evidence standards framework for DHTs [8]. The goal is to assess the available evidence according to the desired evidence standards of an established framework to support decision-making in future implementations of GPS-based alarms.

## Methods

### Protocol and Registration

This systematic literature review was reported according to the PRISMA (Preferred Reporting Items for Systematic Reviews and Meta-Analyses) guidelines [13].

### Eligibility Criteria

The elements of the review (aim, eligibility criteria, and outcomes) were defined as shown in Textbox 1.

**Review elements.**

**Aim**

- To conduct a systematic review of existing evidence of the effects of GPS alarms on the health, welfare, and social provision in the care of older adults compared with standard non–GPS-based care

**Inclusion criteria**

- Original, peer-reviewed publications published between January 2005 and August 2020 in the English language
- Studies performed in Canada, the United States, European Union, Australia, New Zealand, Singapore, Hong Kong, South Korea, or Japan
- Population aged ≥50 years
- GPS-based alarm interventions enabled the users to initiate alarms, with or without localization and with or without geofencing functions

**Exclusion criteria**

- Qualitative studies, technical validations, proof-of-concept studies, system descriptions, reviews, and editorials

**Outcomes**

- Outcomes related to the health and welfare of users and their informal caregivers, and outcomes related to provision in the care of older adults

### Search Strategy

A three-step search strategy was used: (1) an initial search of electronic peer-reviewed scientific publication databases; (2) a search of gray literature databases, trial registers, and Google Scholar; and (3) a search of gray literature from the Nordic countries in targeted websites set up by authorities and organizations, academic publication databases, and Google Scholar. All three steps used a snowballing approach [14,15] in which the articles that reached title-, abstract-, or full-text screening of an initial database search were used in iterations of both backward (reference search) and forward (citation search) snowballing.

### Information Sources and Searches

#### Summary and Complete Strategies

A summary of information sources and retrieved records for all the search steps is presented in Multimedia Appendix 1 [10,16-19]. The complete database-specific search strategies for the initial searches of scientific and gray literature are presented in Multimedia Appendix 2.

#### Scientific Literature

The literature search was completed on August 20, 2020, in the following electronic databases: Academic Search Elite, APA PsycINFO, Applied Social Sciences Index & Abstracts, CINAHL Plus, Cochrane Library, International Bibliography of the Social Sciences, IEEE Xplore, PubMed, Scopus, SocINDEX, Social Services Abstracts, Sociological Abstracts, and Web of Science Core Collection. The search strategy included both free-text and controlled vocabulary. The search was limited to articles published in 2005 or later.

#### Gray Literature

A primary search in gray literature databases was conducted on September 8 and 9, 2020. The databases searched included Base, OpenGrey, OAIster, DART-Europe, ProQuest Dissertations & Theses Global, WHO ICTRP, ClinicalTrials.gov, International HTA Database, and Google Scholar. The search strategy was simplified and adapted to the search interface of gray literature databases. Finally, a larger search for gray literature from the Nordic countries was conducted by searching the websites of government agencies and organizations working with health and welfare issues. Publication databases from universities in the Nordic countries were also searched including the databases and websites of associations of local authorities and regions in the Nordic countries and Google Scholar. The searches were conducted from September 22, 2020, to September 29, 2020, and involved searching for terms in Swedish, Norwegian, Danish, Finnish, and Icelandic.

### Study Selection

The relevant records were downloaded to the reference management software EndNote (Clarivate Analytics). In the search for Nordic gray literature, the retrieved records were screened to determine their relevance related to the aim of the review before downloading the publications to EndNote. In the other searches, all retrieved records were directly downloaded to EndNote. After the removal of duplicates, the records were transferred to the systematic review software Covidence (Veritas Health Innovation Ltd). All four steps were performed by an information science specialist or librarian. The Covidence software, which automatically detected and removed any remaining duplicates, was used for title and abstract screening, full-text review, and data extraction. The screening of titles or abstracts, eligibility assessment of full-text articles, and full-text screening were performed independently by 2 reviewers; any conflicts were resolved by a third reviewer.

### Data Extraction

Information on the included studies’ aims, design, conduct, population, intervention, and outcomes as well as the results for relevant outcomes were extracted from the publications by 2 reviewers independently using a predefined template. Any conflicts in eligibility assessment were resolved through discussion between the reviewers. Information regarding the included studies is presented in Table 1.

### Risk-of-Bias Assessment

Publications that aimed to demonstrate evidence of effectiveness (for tier 3a) were assessed for risk of bias at the study level by 2 researchers independently. The criteria assessed for the randomized study followed the Cochrane Risk-of-Bias Tool 2.0 guidelines [20] and included the method used for random sequence generation, allocation concealment, blinding of participants and personnel, blinding of outcome assessment, the completeness of outcome data, the possibility of selective outcome reporting, and other sources of bias. For nonrandomized studies, the Risk of Bias in Non-randomized Studies of Interventions tool [21] was used to assess bias due to confounding and missing data as well as selective reporting and in the selection of participants and classification of, and deviations from, interventions. Consensus resulted in inclusion of the risk assessment in this review’s summary of findings. Any conflicts in risk assessment were discussed by the 2 researchers (ME and MR) and any conflicts remaining were resolved by the third reviewer (SLS).

### Analysis of Findings From the Systematic Review via the Application of the Evidence Framework

The extracted data for relevant outcomes were categorized and summarized according to the tiers 1-3b evidence categories in the NICE evidence standards framework for DHTs described below. An overview of each study’s contribution to the respective evidence categories is presented in Table 2, and a more extensive version of the table, including the criteria for minimum evidence and best practice standards in all evidence categories, is presented in Multimedia Appendix 3 [22-37]. The table was prepared by one of the researchers and reviewed by a second reviewer. For each evidence category, data extracted from all included studies were compared with the defined minimum evidence and best practice standards of evidence. The extracted data were summarized for each evidence category, and the extent to which the results met the requirements for minimum evidence and best practice standards in each category was assessed. The process was carried out by one of the researchers and reviewed by a second reviewer. Any assessment conflicts were resolved through discussion between the reviewers. The summarized data according to the evidence framework are presented in this review’s summary of the findings (Table 2).

### The Standard Evidence Framework for DHTs

The framework classifies DHTs by function and thereby allows them to be stratified into evidence tiers based on the potential risk to users [8]. Examples of effectiveness and economic value of digital health case studies have been provided to illustrate how the framework can be used [9]. DHTs that track patient location were classified as *active monitoring* and thereby stratified into evidence tier 3b. Moreover, the NICE guidelines state that “best practice evidence standards in each relevant evidence tier should be used for DHTs that present a potential high risk” [8]. GPS alarms can be identified as high-risk DHTs because the intended user group is a vulnerable group and GPS alarm failure could have serious consequences for the user, and in some countries GPS alarms might be used without regular support from social care professionals.

Good levels of evidence for tier 3 DHTs are presented in Multimedia Appendix 3 [22-37]. Evidence standards of the tier 1 and tier 2 categories require documentation that demonstrate that specific aspects have been considered and that the procedures have been completed with a certain level of quality control. Therefore, both the results and aims of the included studies were considered for tier 1 evidence categories. In contrast, tiers 3a and 3b require evidence demonstrating effectiveness.

## Results

### Study Selection

**Figure 1:**
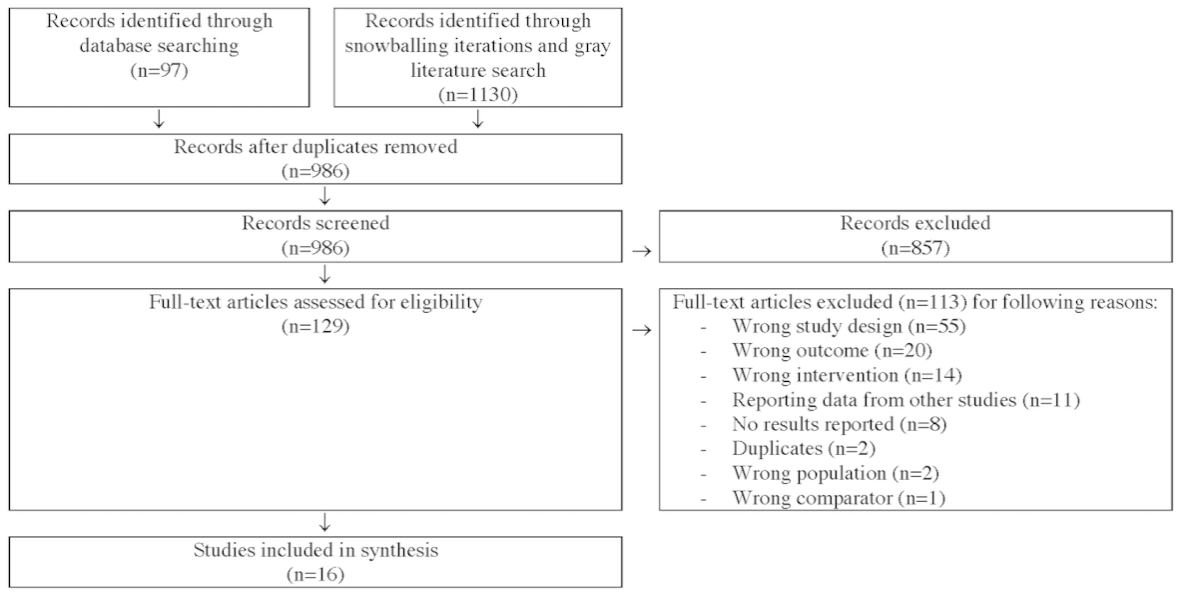
PRISMA (Preferred Reporting Items for Systematic Reviews and Meta-Analyses) flowchart of publications assessed in each step of the review process.

The literature searches identified 1227 records. After the removal of 240 (240/1227, 19.56%) duplicates, the titles or abstracts of 80.36% (986/1227) of the publications were screened for relevance; 69.85%, (857/1227) of articles were excluded in the screening, and 10.51% (129/1227) full-text publications were assessed for eligibility according to the inclusion and exclusion criteria presented in Textbox 1. Of the 129 full-text publications, 113 (87.6%) were excluded, and the remaining 16 (12.4%) articles were included in the final assessment. Figure 1 provides an overview of the publication selection process.

### Study Characteristics

The 16 included studies were published as original articles in scientific journals (n=6), conference papers (n=2), a master’s thesis (n=1), and project reports (n=7). A summary of the characteristics of each publication is presented in Table 1.

Of the 6 peer-reviewed journal studies, 1 was a randomized controlled trial (RCT), and the others were nonrandomized studies with either an experimental (n=4) or an observational design (n=1). The other 10 studies had the following study designs: observational design (n=5, of which 3 used mixed methods and two used quantitative methods), pre- and postintervention design using mixed methods (n=2), economic evaluation (n=2), and within-subjects design using mixed methods (n=1). Of these 10 studies, 5 were part of larger innovation or pilot implementation projects.

The study populations were older adults in general (n=4), older adults with dementia and their family CGs (n=11), and municipal employees working with GPS trackers for persons with dementia (n=1).

**Table 1:** Characteristics of the included studies (N=16).

Study, country	Aim	Intervention	Duration	Design	Participants
Magnusson et al [22], Sweden^a,b^	To investigate views and experiences of persons with dementia living at home, their CGs^c^, and the staff involved in use and implementation of advanced electronic tracking; to analyze results in relation to ethics; and to discuss methodological aspects of research involving persons with dementia	Extended safety and support system Posifon in mobile phone	9 months	Nonrandomized experimental study with pre- and postintervention measurements	Older persons with dementia living at home and their family CGs. Recruited: 76 persons with dementia and 76 CGs; final sample: 20 persons with dementia and 36 CGs
Megges et al [23], Germany^a,b^	To perform an in-depth comparison of the user experiences of persons with dementia and their informal CGs with 2 similar commercial GPS watches in home dementia care; to study the products’ clinical effectiveness in persons with dementia and CGs	2 GPS watches for people with orientation impairment (products A and B)	4 weeks for each product	Nonrandomized experimental study, 2×2 crossover	Dyads: persons with dementia and CGs. Recruited: 12 persons with dementia and 12 CGs; final sample: 4 persons with dementia and 8 CGs
Milne et al [24], Scotland^a,b^	To determine the feasibility of a prospective randomized controlled trial	GPS in watch or pendant, some with a geofencing option. CGs track persons with dementia and are contacted by monitoring agency if persons with dementia report that they are lost or have reached the geofence	4 months (mean and median), range 1-7 months	Mixed methods observational design	20 dyads: persons with dementia with a history of wandering and CGs
Olsson et al [25], Sweden^a,b^	To investigate the effects of using tracking technology on independent outdoor activities and psychological well-being in 3 persons with dementia and their spouses	Passive positioning alarm (transmitter based on GPS, cell phone, and support person)	Intervention phase B1: 5-7 weeks; intervention phase B2: 4-5 weeks	Nonrandomized experimental study with crossover design	3 dyads: persons with dementia and CGs
Pot et al [26], The Netherlands^a,b^	To investigate feasibility, acceptability, and effectiveness of 3-month use of GPS by persons with dementia and CGs	Tracking device (GPS and general packet radio service) worn on belt, including track and trace function, telephone contact, and loudspeaker function	3 months	Nonrandomized experimental study	Dyads: persons with dementia and CGs; persons with dementia in early stage of dementia. Recruited: 33; included: 28
Scheffer et al [27], The Netherlands^a,b^	To investigate the effects of a mobile safety alarm on frequency of going outside and experiences regarding fear of falling, feelings of being unsafe, and quality of life in older adults	Mobile safety alarm with a built-in drop sensor using a positioning system over a mobile phone network	6 months	Randomized controlled trial	Older adults using indoor alarm. Included: 203 (100 in intervention and 103 in control); final sample: 135 (58 in intervention and 77 in control)
Ribas Miquel et al [37], Spain^d^	To describe the perceptions and experiences of professionals and family members of users and nonusers of GPS tracking devices	GPS tracking device	N/A^e^	Mixed methods observational design	30 care professionals, 7 family members of care receivers who use a GPS tracking device, and 7 family members of care receivers not using a tracking device
Røhne et al [28], Norway^b,f^	To verify if or how mobile safety alarms make older adults more independent, increase their mobility and physical activity, engage relatives and lead to reduced need for care, and increase the ability of older adults to live longer at home	Global System for Mobile communication or GPS-based alarm unit (hanging around the user’s neck) with geofencing, voice connection, and tracking ability	2-9 months	Mixed methods with pre- and postintervention design^f^	Populations of older adults in Bærum, Skien, and Stavanger municipalities. Included in pilot: 71; included in evaluation: 46
Sørli [29], Norway^g^	To investigate the effects and the experiences of municipal employees of GPS use among persons with dementia on the quality of the municipal services	GPS tracking device	8 months	Observational design	19 municipal employees working with GPS for persons with dementia
Ausen et al [30], Norway^h^	To establish knowledge about users’ and employees’ experience of piloted GPS; to establish knowledge of the effects of use of the technology on users, employees, and the service; to identify potential gains; to describe service models for the use of various security and coping technologies in the municipality	GPS unit (possible to attach to key chain) for localization with geofencing alarm	1 year (mean)	Observational design^f^	Older adults living at home or in nursing homes in Larvik. 47 care receivers; number of employees unclear
Boysen and Støle [31], Norway^h^	To establish knowledge of the users’ and employees’ experience of the technologies being piloted; to identify potential gains; and to propose measures to realize the benefits	Mobile safety alarm with GPS tracking and geofencing (Safemate)	6 months	Mixed methods with pre- and postintervention design^f^	9 persons with dementia and with affiliation with either housing associations, nursing homes, or activity centers; 13 home users with follow-up of relatives
Dahlberg [32], Sweden^h^	To perform a socioeconomic analysis in the form of a cost-benefit assessment of a mobile security alarm	Mobile safety alarm with GPS tracking, geofencing, and voice communication (Posifon, same as [22])	9-10 months	Economic evaluation	Persons with mild and more advanced dementia; persons with more advanced dementia with municipal care; all living in a home setting with some form of care from relatives. Approximately 80 (same as [22])
Malmquist [33], Sweden^h^	To assess the costs and benefits of passive alarms for users, their relatives, the municipality, and society; to develop a decision-making basis for prescription of different passive alarms; to increase knowledge regarding passive alarms and their costs and benefits for stakeholders	GPS or Global System for Mobile communication passive position alarm (bracelet) with geofencing function	37-260 days	Economic evaluation	8 persons with dementia living in home settings in Östersund municipality and their relatives or informal CGs. Prescribers of alarms within the municipality
Øderud et al [34], Norway^h^	To investigate how location technology can be organized and integrated as part of the municipalities’ operational health and care services and to develop service models for interaction among public, private, and volunteer service providers to help persons with dementia and their CGs	GPS devices (1 of 3) with alarm, tracking, and voice communication features	Up to 1 year (58% of the users); 1-2 years (35% of the users), more than 2 years (7% of the users)	Mixed methods observational design^i^	Oslo inhabitants with dementia or dementia-like conditions, 94% living in their own home. 109 users; 216 in total (users and their relatives, employees in care services and at alarm center, and localization technology providers)
Røhne et al [35], Norway^h^	To investigate how mobile safety alarms can contribute toward enabling older adults to reside at home for as long as possible and to establish knowledge of how today’s mobile safety alarms can be developed	Mobile safety alarm with tracking and voice communication	8-10 months	Mixed methods observational design^j^	Older adults living independently Recruited: 12; final sample: 10
Vidensförmidling and Syd [36], Denmark^h^	To investigate whether early allocation of GPS alarms can provide greater security and quality of life for persons with dementia and their relatives and thereby reduce the need for help from the municipality	A combined GPS or Global System for Mobile communication device carried in a pocket or worn on the belt, individually adapted to the individual. Unit equipped with a call button, with which the person with dementia can call for help in the event of a fall or similar incident	At least three months	Mixed methods, within-subjects design; economic evaluation^k^	180 persons with dementia and their informal CGs living in the home setting in 5 municipalities

^a^Journal article.

^b^Peer-reviewed.

^c^CG: caregiver.

^d^Conference paper.

^e^N/A: not applicable.

^f^Part of a larger project that, according to the publication, used methods from research-supported, demand-driven innovation.

^g^Master’s thesis.

^h^Project report.

^i^Part of a larger project which, according to the publication, used methods from demand-driven innovation and service design to go from pilot to operation.

^j^Part of pilot implementation study.

^k^Part of demonstration project.

### Summary of Findings

Table 2 presents an overview of the study findings from individual studies assessed using the NICE evidence standards framework for DHTs [8]. A more extensive version of the table, including the definition of minimum evidence and best practice standards for each category, is presented in Multimedia Appendix 3 [22-37]. As can be seen in Table 2, the included studies provide best practice evidence according to the standards of two of the five tier 1 evidence categories, that is, *Relevance to current pathways in health/social care system* and *Acceptability with users*. In more than 60% of the included studies, GPS devices had been successfully piloted or implemented in social care systems. All these studies had been performed in Nordic countries as part of larger projects supporting the development of products, services, and decision-making processes. Most of these projects were part of national government programs that aimed to stimulate the use of welfare technology. Furthermore, best practice evidence showing that representatives from the intended user groups (older adults and persons with dementia) were involved in the testing of the GPS alarms and that the users were satisfied with them was identified in 38% (6/16) of the included studies. Table 2 also shows that minimum standard evidence was identified for the tier 1 category *Credibility with health*, *social care professionals* because relevant social care professionals had been involved in 75% (12/16) of the included studies.

However, the evidence according to the standards for tier 2 evidence categories could not be assessed from the included studies. For example, because the alarm systems do not provide general information or advice to users concerning health, healthy living, lifestyle, diseases, illnesses, or conditions, the minimum and best practice standards were not relevant for this assessment. Nevertheless, the information that the alarm systems provides to formal and informal CGs and security service providers about user position and emergency situations must be accurate. None of the evaluated studies investigated the reliability of this information. However, one study elaborated on alarm testing and the timeliness of the transmission of information in case of an alarm during the development of test routines, and two studies investigated the CGs’ views on the accuracy of the information regarding the user position and the user-friendly aspects of the interfaces. The two latter studies identified that some situations (eg, when users reach places with poor mobile coverage) can limit the updating of user position and thereby reduce the reliability of the system’s information content.

Furthermore, evidence of ongoing data collection to show use and value could not be identified from the included studies. Indeed, several studies demonstrated use and value on study follow-up occasions. Use was reported both for specific system functions and on a system level with varied levels of detail. Although some studies collected use data from system logs, reporting by CGs or users was more common. The measured values for users (persons with dementia or older adults) related to improved outdoor activity (increased independence, fewer worries, increased frequency with regard to visiting new places and making longer trips, and increased or maintained physical activity level); improved relationship with CGs (fewer conflicts with CGs and more freedom); increased security and safety (increased security in daily life and prolonged period living independently); and increased QoL. The measured values for CGs of persons with dementia related to improved well-being, QoL, enhanced possibility of giving more freedom to persons with dementia, and improved security and safety. Finally, examples of measures for safeguarding (service models, test routines, and role of relatives or alarm center) in the use of GPS alarms were described in three of the included studies. However, evidence according to the standards could not be identified.

Moreover, the included studies provide clear evidence of effectiveness in outcomes or improvements in outcomes relevant to tiers 3a and 3b. Of the 16 included studies, 10 [22-27,32,33,36,37] investigated the outcomes that could be related to effectiveness. The findings of the included RCT [27] were compared with the framework’s standards for best practice evidence to demonstrate effectiveness in outcomes or improvements in outcomes. However, no increase in the frequency of older adults going outside was found in the intervention group, and no signiﬁcant differences in secondary outcomes, including fear of falling, feelings of unsafety, or QoL, were identified [27]. The other nine studies that used observational or quasi-experimental designs were assessed with regard to requirements for minimum evidence standards (ie, demonstrating effectiveness in outcomes or improvements in outcomes). Improvements were indicated with regard to an increase in the percentage of days that persons with dementia were engaged in independent outdoor activity (there were indications of an increase in three cases based on CG reports; no statistical data were available) [25] and to a decrease in role-overload and feelings of worry for CGs of persons with dementia (*P*>.05) [26]. Furthermore, GPS tracker use was associated with an important decrease in time spent searching for persons with dementia who were lost [24]. However, the data were based on CG recall and could not be objectively verified. In addition, this outcome is more related to efficiency than effectiveness. Interestingly, one study found decreased activity among persons with dementia because of disease progression [22], and another study identified no significant changes in burden or QoL for CGs of persons with dementia [23]. In contrast, economic evaluations indicated reduced costs for the care of persons with dementia because of prolonged periods living independently instead of special housing (up to 3 months) [32,33,36]. As a crossover design was used, the difference in the mean CG burden between relatives of persons with dementia using or not using GPS trackers was indicated in small samples [37].

**Table 2:** Summary of findings in relation to the evidence categories of the evidence standards framework for digital health technologies (N=16).

Tiers			Findings		Studies, n (%)		Risk of bias^a^
**Tier 1**							
	Credibility with health and social care professionals	Minimum evidence standards show that relevant social care professionals were involved in the design, development, or testing of the GPS devices In 12 (75%) [22,24,25,28-36] of the included studies, social care professionals were involved in the testing of the GPS devices to a varied extent		12 (75)		—^b^	
	Relevance to current pathways in health or social care system	Minimum and best practice evidence standards show that GPS devices have been successfully piloted or implemented in social care systems. This was described in 10 (63%) of the included studies. Of these 10, 3 were performed in Sweden [22,32,33], 6 in Norway [28-31,34,35], and 1 in Denmark [36]. All of them were part of larger projects supporting development of products, services, and decision-making processes to support OAsc and their families in their homes. Most of those projects were part of national government programs that aimed to stimulate the use of welfare technology		10 (63)		—	
	Acceptability with users	Best practice evidence shows that representatives from the intended user groups (persons with dementia and OAs) were involved in the design, development, or testing of the DHTd and to show that users were satisfied with the DHT Representatives from the intended user groups (OAs in general or persons with dementia) were involved in testing of the GPS alarms in 15 (94%) of the studies (ie, all the included studies except [29]) Six (38%) of these studies showed that the users were satisfied with the alarms: 77% of the CGse of persons with dementia stated that they would recommend the use of GPS alarms in the Pot et al study [26]; 97% of the OAs who participated in the Røhne et al study [28] and 90% of the OAs in the Røhne et al study [35] stated that they were satisfied with the alarm All older users in the Ausen et al study [30] would recommend others in similar situations to use the GPS alarm User satisfaction was confirmed in the interviews in the Milne et al study [24] and in the values identified in the Boysen et al study [31]		6 (38)		—	
	Equalities considerations	No information retrieved from included studies. Socioeconomic aspects were not addressed However, persons with dementia and OAs may be considered vulnerable groups		0 (0)		—	
	Accurate and reliable measurements (if relevant)	No information retrieved from included studies. Technical validations were not included in the review. All included studies used commercial products		0 (0)		—	
	Accurate and reliable transmission of data (if relevant)	No information retrieved from included studies. Technical validations were not included in the review. All included studies used commercial products		0 (0)		—	
**Tier 2**							
	Reliable information content	Minimum and best practice standards category is not relevant for GPS devices because they do not provide general information or advice to users concerning health, healthy living, lifestyle, diseases, illnesses, or conditions However, for reliability of information on user position and emergency situations, data on user testing were provided by 1 study (6%) [28], and data on CGs’ perceptions of the accuracy of the GPS information were provided by 2 (13%) studies. For example, relatives and staff in the Øderud et al study [34] had experienced slow or unreliable information on the user’s position. Moreover, [30] reported cases of poor mobile coverage that had resulted in failures in updating user position		0 (0)		—	
	Ongoing data collection to show use	Cannot be assessed from the included studies. Evidence of ongoing data collection (required according to evidence standards for the category) was not reported in the included studies However, 10 (63%) of the included studies presented data on use on specific occasions related to the interventions In all, 3 studies (19%) reported quantitative data on usage period: [30] and [34] presented the number of participants who had used GPS trackers for up to 1 year and between 1 and 2 years, respectively. [33] reported the number of days that each participant had used the GPS trackers (mean 158 days, median 210 days, and range 37-260 days) A total of 3 (19%) studies [22,25,28] included system logs in the collection of data to investigate use. Interestingly, [22] saw that the extent to which persons with dementia used mobile phone–based GPS varied widely among the participants. Moreover, [28] described that the logs from the technical systems were thoroughly analyzed to understand the role and function of users, alarm units, response center, CGs, and relatives In all, 4 (25%) studies [23,24,26,27] based the data collection of use on the recall of the users or their CGs, and 2 of these [26,27] reported that the persons with dementia did not always take along the GPS devices (mobile phone or tracker worn on the belt) when going out and that the devices were not always switched on		0 (0)		—	
	Ongoing data collection to show value	Cannot be assessed from the included studies. Evidence of ongoing data collection to show value (required according to evidence standards for the category) was not reported However, 11 (69%) of the included studies presented data on use for values related to the health and welfare outcomes of users (OAs, persons with dementia, and CGs of persons with dementia) on specific occasions related to the interventions; one (6%) study [22] identified that CGs experienced that the persons with dementia had become more independent in outdoor activity; 1 (6%) study [24] identified that CGs and staff saw that GPS trackers could give persons with dementia in milder stages of dementia and their CGs increased freedom and decreased stress and anxiety; and 1 (6%) study [26] identified values perceived by some of the CRsf, including increased freedom and decreased worries and fewer conflicts with CGs when going outside alone. Moreover, the CGs experienced that they gave more freedom to the CR and some experienced fewer conflicts with the CR Another study (6%) [28] identified that more than 50% of the users thought that the GPS alarm helped to increase their freedom One study (6%) [29] noted that more than 50% of the participating staff perceived that GPS trackers for persons with dementia could, to some degree, free up time for service providers by reducing the number of inspections they carried out to see if the person is well, driving to and from the user and following the user on walks; [30] identified that all persons with dementia thought that GPS trackers enabled them to increase or maintain physical activity, to increase freedom in outdoor activities, and that all relatives experienced that the GPS trackers increased their feelings of safety when leaving the person with dementia by themselves; and [31] identified positive values of GPS trackers both in shared housing for persons with dementia, including freedom for persons with dementia, decreased stress and anxiety for employees, time savings for staff and cost reduction, and for home users, including increased security, with, in some cases, increased outdoor activity and CG relief Another study [32] identified that 5 of the 8 GPS tracker users experienced increased security and could continue to live at home for a longer periods. In addition, 5 of the 8 relatives experienced fewer concerns and worries; [34] noted that most of the users perceived that the GPS trackers provide security (for the user, CG, and staff), increase freedom for the user and sometimes also the CG, as well as help the user to be physically active and maintain their activity level; [35] identified that most of the GPS alarm users experienced that it increased their safety and freedom in daily life; and [36] identified that the GPS tracker increased the security and quality of life of persons with dementia and their CGs		0 (0)		—	
	Quality and safeguarding	Cannot be assessed from the included studies However, 3 (19%) of the included studies had a study aim or presented data related to system-level quality and safeguarding: [30] and [34] presented the service model for implementation of GPS trackers in the homes of older adults, which included safeguarding measures taken by the municipality. Moreover, [34] presented data on the roles of different actors (users, relatives, and alarm centers) in charging and administration of the alarm as well as locating and retrieving the user, if necessary One study (6%) [28] described the establishment of an initial test routine to encourage users to regularly trigger the alarm when out walking		0 (0)		—	
**Tier 3a**							
	Demonstrating effectiveness in outcomes or improvements in outcomes	Effectiveness is not demonstrated in outcomes or improvements in outcomes according to best practice standards: no increase in the frequency of OAs going outside; no signiﬁcant differences in changes in fear of falling, feelings of unsafety, or quality of life [27] Effectiveness is not demonstrated in outcomes or improvements in outcomes according to minimum evidence standards. Indications were identified for the following: Decrease in time searching for person with dementia (from a mean of 3-4 hours per event to 40 minutes) [24] Increase in the number of days that person with dementia was engaged in independent outdoor activity (three cases, no statistics available) [25] Decrease in role-overload of CGs of persons with dementia (*P*=.126; d=–0.25 for all CGs, and *P*=.119; d=–0.34 for CGs who could reach CR with the mobile alarm) and in feelings of worry (*P*=.08; d=–0.32 for all CGs, and *P*=.057; d=–0.46 for CGs who could reach CR with the alarm) [26] Reduction in costs for care of persons with dementia because of prolonged time that the person could live independently instead of in special housing (up to 3 months) [32,33,36] Difference in mean CG burden between relatives of persons with dementia using and not using GPS (*P*=.04) was indicated in small samples because a crossover design was used [37] Outcomes investigated with negative results: Activity of person with dementia: reduced because of disease progression [22] Burden and quality of life for CGs of persons with dementia: no significant changes [23]		1 (6) 7 (44) 2 (12)		High High High	
	Use of appropriate behavior change techniques (if relevant)	Not relevant for GPS devices: no behavior change techniques used		0 (0)		—	
**Tier 3b**							
	Demonstrating effectiveness: improvements in outcomes	Effectiveness is not demonstrated in improvements in outcomes according to minimum evidence or best practice standards (see above)		0 (0)		—	

^a^Risk-of-bias assessment: a “high” risk of bias is equivalent to “high” for the Cochrane Risk-of-Bias Tool 2.0 (randomized studies) or “serious/critical” for the Risk of Bias in Non-randomized Studies of Interventions Tool (nonrandomized studies), and a “low” risk of bias is equivalent to “low” for the Cochrane Risk-of-Bias Tool 2.0 (randomized studies) or “low/moderate” for the Risk of Bias in Non-randomized Studies of Interventions Tool (nonrandomized studies).

^b^Not available.

^c^OA: older adult.

^d^DHT: digital health technology.

^e^CG: caregiver.

^f^CR: care receiver.

### Risk of Bias

The summaries of the assessed risk of bias for individual studies that aimed to demonstrate evidence of effectiveness are presented in Table 3 (nonrandomized studies) and Table 4 (randomized study). The overall risk of bias was assessed to be serious or critical in all nonrandomized studies, particularly bias due to confounding and in measuring outcomes [22-26,32,33,36,37]. The RCT was assessed as having a high risk of bias, in particular with regard to the blinding of patients or personnel and incomplete outcome data [27].

**Table 3:** Risk-of-bias assessments for individual studies investigating effectiveness according to standardized criteria for nonrandomized studies using the Risk of Bias in Non-randomized Studies of Interventions Tool. The tool’s scale for ascending risk is low, moderate, serious, and critical.

Domains of bias	Study								
	Magnusson et al [22]	Megges et al [23]	Milne et al [24]	Olsson et al [25]	Pot et al [26]	Ribas Miquel et al [37]	Dahlberg [32]	Malmquist [33]	Vidensförmidling and Syd [36]
Bias due to confounding	Serious	Serious	Critical	Moderate	Serious	Serious	Critical	Critical	Critical
Bias in selection of participants	Low	Low	Critical	Serious	Low	Serious	No information	Serious	No information
Bias in classification of interventions	Low	Low	Low	Low	Low	Moderate	No information	Moderate	Moderate
Bias in deviations from intervention	Low	No information	Low	Low	Serious	No information	Serious	No information	No information
Bias due to missing data	Critical	Moderate	Serious	Low	Moderate	No information	No information	Low	No information
Bias in measurement of outcomes	Serious	Serious	Serious	Serious	Serious	Serious	Moderate	Critical	Serious
Bias in selective reporting	Low	Moderate	Serious	Low	Low	Low	Serious	Serious	Serious
Overall bias	Critical	Serious	Critical	Serious	Serious	Serious	Critical	Critical	Critical
Comments or direction of bias	Unpredictable	Unpredictable	Unpredictable	Unpredictable	Unpredictable	Unpredictable	Unpredictable	Unpredictable	Unpredictable

**Table 4:** Risk-of-bias assessments for individual studies investigating effectiveness according to standardized criteria for randomized studies using the Cochrane Risk-of-Bias Tool 2.0. Bias is assessed as low, high, or unsure (when sufficient information is not available to allow assessment).

Risk of bias domain	Study (Scheffer et al [27])
Random sequence generation	Low
Allocation concealment	Low
Blinding of participants or personnel	High
Blinding of outcome assessment	Unsure
Incomplete outcome data	High
Selective reporting	Low
Other bias	Unsure
Comments	High dropout, especially in the intervention group where the provided foremost reason was that the participants found the alarm too big and heavy

## Discussion

### Principal Findings

GPS alarms are implemented in social care with the aim of supporting users in independent activities of daily living, particularly outdoors. This systematic review included 16 studies investigating the effects of GPS alarms on health, welfare, and social provision in older adult care. The review demonstrates best practice evidence in peer-reviewed and gray literature for two of the tier 1 evidence categories (*Relevance to current pathways in health/social care system* and *Acceptability with users*) of the NICE evidence standards framework for DHTs [8]. The approach of using an existing framework to assess the quality of studies is relevant for all types of DHTs. This review also presents evidence of the minimum standard for the tier 1 category *Credibility with health, social care professionals*, although the minimum standard is insufficient for high-risk DHTs such as technologies that track patient location. More specifically, the studies in this review showed that social care professionals have been involved in the testing of alarm systems; that GPS trackers have been successfully piloted or implemented in the Swedish, Norwegian, and Danish social care systems; that representatives from the intended user groups (persons with dementia and older adults) have been involved in testing the alarm systems; and that users were satisfied with the alarm systems. Although a number of studies reported findings regarding use, value, and measures for safeguarding at specific time points, our review concluded that evidence categories for tier 2 could not be assessed from the included studies. Finally, this review identified a lack of clear evidence for effectiveness according to the standards of evidence categories in tiers 3a and 3b in the NICE framework, which is required for DHTs that track patient location [8]. Moreover, the overall risk of bias of the included studies that evaluated effectiveness was assessed to be high. Therefore, the study findings should be interpreted with caution.

Some of the included studies demonstrated negative or absent effects on healthy behaviors and on the users’ health and welfare. For example, the only RCT included in our review demonstrated no effect of GPS trackers on older adults’ frequency of going out, feelings of unsafety, or fear of falling, and the authors stated that “some of the participants did not take the mobile alarm outside with them at all times. This might have been caused by perceived user‐unfriendliness of the alarm [27].

Moreover, a nonrandomized intervention study demonstrated that activity among persons with dementia was reduced during the intervention and concluded that this was due most likely to disease progression [22]. The need to identify primary user groups has been addressed in previous research on GPS alarms for older adults [10]. Both examples illustrate the complexity of this type of intervention in populations of persons with dementia, which might increase the risk of bias due to deviations from the interventions and to missing data, respectively. Additional potential challenges for RCTs investigating the use of GPS alarms in persons with dementia were identified in a feasibility study [24] and included challenges in finding social care staff willing to recruit participants, randomizing the participants, and finding participants or CGs willing to participate as controls [24].

It should be pointed out that GPS trackers and the context of use varied among the studies included in this review; simply put, these studies evaluated different wearables, technical infrastructures, and supporting services. For example, some studies reported on insufficient usability of the trackers, which might have limited their use. However, the rapid development of mobile health systems has enabled the incorporation of GPS alarms in discrete wearables such as bracelets and pendants or consumer products that older adults are already using in daily life. These more sophisticated wearables might help to overcome the obstacles of poor usability and the potential stigma of older GPS devices such as mobile trackers attached to the belt or additional mobile phones. Hence, the usability, acceptability, and desirability of GPS wearables need to be addressed in future research [38]. Other aspects that are relevant to investigate in future studies of GPS use include the system’s ease of use (including usability and learnability) and the users’ readiness (including expectations [39] and eHealth literacy [40]). In addition, involving user groups in the design of user interfaces and supporting services can be effective in the development of new systems that meet the needs of user groups and the context of use [41,42].

The alarm systems studied have been implemented to varying degrees in the social care systems of different countries. User and CG support services also varied across the studies: in some cases, CGs were the sole alarm receivers, whereas in others, resources from a security company or the municipality have supported the CGs.

Interestingly, economic effects are likely to be affected by the value models and organization of national social care systems. In this review, the heterogeneity of the studies in terms of population, context, and systems was high; therefore, the generalizability of the results is uncertain.

Further research on service development, implementation of information and communication technologies in the public sector, profit realization, processes for change, contribution to political goals, business models, and health and welfare has been recommended to support the implementation of GPS alarms in older adult care [10]. Studies in this review that were found in gray literature have demonstrated progress in these areas (eg, usable and acceptable systems that were properly integrated in the social care services of Nordic countries). These studies were conducted as a part of larger projects—often within national programs to promote the use of HWTs among older adults—that aimed to study and develop products, services, and methods for implementation.

Notably, the studies included in this review identified outcomes that might be relevant for future studies investigating the effectiveness of GPS-based mobile alarms on the health and welfare of older adults and social care provision. Furthermore, outcomes related to the health and welfare of CGs of persons with dementia might be relevant for further investigation [23,26]. Other outcomes related more to efficiency, such as time spent searching for persons with dementia who were lost [24], may be less useful in assessing effectiveness and should therefore be considered as complementary or secondary.

This review identified a need to establish evidence in several evidence categories in all tiers of the NICE evidence framework. Evidence for tiers 1 and 2 might be identified from several sources, including product documentation from suppliers of HWT products; expert authorities; and initiatives for service development, piloting, and implementation of HWT products. However, for tiers 3a and 3b, further research on the effectiveness of GPS-based mobile alarms is still needed.

### Limitations

A potential limitation of this review is the exclusion of studies with a qualitative design as well as technical validation studies. The findings from these types of studies might have been relevant for providing evidence for tiers 1 and 2. The study findings were assessed using the NICE evidence standards framework for DHTs [8], which includes evidence categories (especially in tiers 3a and 3b) that do not support qualitative studies. However, other evaluation strategies exist [43]. In addition, the searches were restricted to the English and Nordic languages, thus excluding studies published in other languages.

### Comparison With Prior Work

The findings of this review are in line with previous reviews of GPS tracker use among older adults (including persons with dementia), which identified limited evidence on the effectiveness of GPS tracker use on health-related outcomes, for example [10-12]. The previous reviews provided a broader picture of state-of-the-art research on GPS tracker use in older adult care [10] and for managing wandering behavior among persons with dementia [11]. For example, the study by Røhne et al [10] categorized findings in different research areas from 74 articles published from 1998 to 2016. The study by Røhne et al [10] concluded that research on GPS had increased significantly by 2014-2015, most likely because of the increased use of smartphones, wearables, and other health technologies, and had concentrated, up to that time, on identifying the primary user groups and their needs and experiences. Moreover, the study by Røhne et al [10] identified knowledge gaps related to service development, implementation of information and communication technologies in the public sector, profit realization, change processes, contributions to political objectives, business models, and health and well-being that need to be filled for location services to be used as ordinary services in Norwegian municipalities. This systematic review could not identify a large increase in published studies of the effectiveness of GPS alarms on health, welfare, and social care outcomes after 2014. On the contrary, the publication years of the studies included in this review are rather evenly distributed over the search period (two each in 2012 and in the years 2014-2017, one each in 2011 and 2018, and four in 2013). It is worth mentioning that the included economic evaluations were published in 2011 and 2013.

Moreover, the scoping review by Neubauer et al [11], which included studies that used any type of study design or methodology with positive or negative results, identified that the most commonly used wander management technology—from scholarly and gray literature—was GPS, followed by alarms and sensors, with only 22% of the devices clinically tested in home or institution settings. The review by Neubauer et al [11] concluded that further research is needed to identify technologies with high levels of evidence for effectiveness and usability. The seven studies on mobile locators included in the review by Neubauer et al [11] were published in the period 2000-2012. Interestingly, no overlap in the included studies was identified between the review by Neubauer et al [11] and this review. However, this review had a small overlap of four studies with a synthesizing review by Bartlett et al [12], which included empirical studies of persons with dementia or their family CGs or both using GPS. A total of 23 studies published in the period 2007-2016 were included, and data were synthesized across three identified themes: using GPS to prevent harm and promote well-being; taking control; and value of GPS data [12]. The review by Bartlett et al [12] found only nontrial evidence and identified a lack of large-scale studies. This review therefore complements the previous reviews in several aspects: first, by analyzing data from different studies, mainly because of the inclusion of only quantitative results; second, by contributing results published after 2016; and third, by reviewing the current evidence on GPS trackers according to the requirements of the NICE framework. So far, the NICE framework has been used for examples of case studies that demonstrate evidence of the effectiveness and economic value of a number of DHTs [9]. These studies are based largely on information provided by the developers that has not been independently verified. Study results published in peer-reviewed and gray literature can therefore strengthen the validity of evidence of DHTs. We have not been able to identify prior systematic reviews of evidence related to HWTs that have applied the NICE framework to the study findings. However, one study has used the framework for classifying medical mobile apps [44]. We envision that the number of studies that apply the NICE framework for various purposes will increase in the future and contribute to demonstrating evidence related to HWTs.

### Implications for Clinical Practice and Future Research

This review has identified a need for further research to provide the required evidence for the effects of the use of GPS-based mobile alarms on the health and welfare of older adults and social care provision. On the basis of the NICE evidence standards framework, examples of successful piloting of GPS alarms in social care systems and of testing that involved older adults and CGs have been identified.

The results can be beneficial to the social care organizations that see the potential in GPS alarms to support older citizens’ independence in daily living activities. First, we demonstrated that there is a lack of evidence of the clinical effectiveness of GPS trackers in the care of older adults. Hence, the ongoing implementations of GPS trackers have not been based on evidence of their clinical effectiveness. Second, we clarified the risks of implementing patient-locating systems such as GPS with insufficient evidence based on the NICE evidence framework. On the basis of the framework, the types of potential risks associated with the use of HWTs with insufficient evidence (eg, safety risks for the user and inefficient use of resources) can be identified. Social care organizations can use this knowledge to make informed decisions on whether they should wait for, or demand, more evidence before they start using new HWTs. Hence, increased awareness of what can be gained from better evidence is important. Third, we presented experiences from successful piloting of GPS trackers in social care systems, for example, with regard to suitable user groups, usable and acceptable GPS solutions, customization and development of products and services, processes for decision-making, and implementation. The level of evidence regarding effectiveness and economic value needs to be considered in decision-making processes on the implementation of HWTs when potential benefits and risks need to be balanced. After all, money spent on improving the health of older adults needs to be spent wisely and efficiently.

This review has identified critical knowledge gaps that need to be addressed in future research, most importantly with regard to clinical effectiveness. Open questions include the clinical effectiveness of specific GPS trackers for certain user groups with defined supporting services. The included studies can contribute with positive examples of implementation on which to base future research (eg, products, user groups, and service models) and with challenges that have been experienced in previous research. Potential evolutions of this review are future studies on the clinical effectiveness of already implemented or piloted GPS trackers in their *real-world* implementation environment. Thus, the interventions to be evaluated should include user groups and service models that have been identified and developed in the previous implementation or pilot projects. In addition, generating real-world evidence during the implementation of GPS trackers in social care systems seems to be of great importance in addressing the evidence gaps.

### Conclusions

There is insufficient evidence for the effects of the use of GPS-based mobile alarms on the health and welfare of older adults, as well as social care provision. Best practice evidence for two of the tier 1 evidence categories and evidence of minimum standard for the tier 1 category of the NICE evidence standards framework for DHTs [8] were identified. This review identified that social care professionals have been involved in the testing of GPS trackers, that GPS trackers have been successfully piloted or implemented in the Nordic social care systems, that representatives from the intended user groups (persons with dementia and older adults) have been involved in testing the devices, and that users were satisfied with them. Although a number of studies have contributed data demonstrating use, value, and measures for safeguarding at specific time points, our review concluded that the evidence categories for tier 2 could not be assessed from the included studies. Finally, this review demonstrated a lack of clear evidence of effectiveness according to the standards of evidence categories in tiers 3a and 3b in the NICE framework, which is required for DHTs that track patient location [8]. Future research needs to address clinical effectiveness broadly and incorporate aspects related to products, user groups, service models, and challenges in social care systems in the *real world*.

## Appendix

Multimedia Appendix 1 Overview of the search process and the results.

Multimedia Appendix 2 Complete database-specific search strategies for the initial searches of scientific and gray literature.

Multimedia Appendix 3 Study findings related to the requirements of minimum evidence and best practice standards of each evidence category in the NICE evidence standards framework. NICE: National Institute for Health and Care Excellence.

## Abbreviations

CG: caregiver
DHT: digital health technology
HWT: health and welfare technology
NICE: National Institute for Health and Care Excellence
PRISMA: Preferred Reporting Items for Systematic Reviews and Meta-Analyses
QoL: quality of life
RCT: randomized controlled trial
