# Supplementary material for: Mobile Safety Alarms Based on GPS Technology in the Care of Older Adults: Systematic Review of Evidence Based on a General Evidence Framework for Digital Health Technologies
Source: J Med Internet Res. 2021 Oct 11;23(10):e27267. doi: 10.2196/27267 (PMC8546532; doi:10.2196/27267)
Supplement: Multimedia Appendix 1 [file jmir_v23i10e27267_app1.docx]

| Search steps | Sources | Number of retrieved publications |
| --- | --- | --- |
| 1. Initial search in scientific databases | Databases:  Academic Search Elite (EBSCOhost), APA PsycInfo (EBSCOhost), Applied Social Sciences Index & Abstracts – ASSIA (ProQuest), Cinahl Plus (EBSCOhost), Cochrane Library (www.cochranelibrary.com), International Bibliography of the Social Sciences – IBSS (ProQuest), IEEE Xplore ( https://ieeexplore.ieee.org), PubMed (https://pubmed.ncbi.nlm.nih.gov/, Scopus (www.scopus.com), SocINDEX (EBSCOhost), Social Services Abstracts (ProQuest), Sociological Abstracts (ProQuest), Web of Science Core Collection (www.webofknowledge.com). | 68  (29 duplicates removed) |
| 2. Snow-balling iteration 1 | Databases:  Scopus ([1*], [2*]) and Google scholar ([3*]) | 288  (34 duplicates removed) |
| 3. Snow-balling iteration 2 | Relevant references of 9 review articles from previous iteration | 102  (1 duplicate removed) |
| 4. Initial search in grey literature databases, trial registers and Google Scholar | Databases:  Base (www.base-search.net), Google Scholar (https://scholar.google.com), Open Grey (www.opengrey.eu), OAIster (https://oaister.worldcat.org), DART-Europé (www.dart-europe.eu), ProQuest Dissertations & Theses Global (ProQuest), WHO ICTRP (https://apps.who.int/trialsearchClinicalTrials.gov (www.clinicaltrials.gov), International HTA database (www.inahta.org/hta-database/) | 115  (19 duplicates removed) |
| 5. Snow-balling iteration 3 | Selected references from [10] and [4*] | 5 |
| 6. Larger search for grey literature (Nordic countries) | Publication sites owned by universities and colleges:  SwePub (http://swepub.kb.se/)  CRISTIN (https://app.cristin.no/ /)  Forskningsdatabasen ([www.forskningsdatabasen.dk](http://www.forskningsdatabasen.dk)),  Publikationsportalen Juuli ([www.juuli.fi](http://www.juuli.fi))  Opin visindi (https://opinvisindi.is/)  Municipalities’, countries’ and regions’ central organisations:  Sveriges Kommuner och Regioner ([www.skr.se](http://www.skr.se))  Kommunernes Landsforening ([www.kl.dk/](http://www.kl.dk/))  Kommunesektorens organisasjon ([www.ks.no/](http://www.ks.no/))  Kommunforbundet ([www.kommunforbundet.fi](http://www.kommunforbundet.fi))  Icelandic Association of Local Authorities ([www.samband.is/english/](http://www.samband.is/english/))  Organisations responsible for elderly care, digitalization in health and welfare, and related issues  Socialstyrelsen ([www.socialstyrelsen.se/](http://www.socialstyrelsen.se/))  The Directorate of Health, Iceland ([www.landlaeknir.is/english/](http://www.landlaeknir.is/english/))  Socialstyrelsen (https://socialstyrelsen.dk/)  Digitaliseringsstyrelsen (https://digst.dk/  Helse- og omsorgsdepartementet ([www.regjeringen.no/no/dep/hod/id421/](http://www.regjeringen.no/no/dep/hod/id421/))  Helsedirektoratet, ([www.helsedirektoratet.no/](http://www.helsedirektoratet.no/))  Direktoratet for e-helse (https://ehelse.no/  Folkhelseinstituttet ([www.fhi.no/](http://www.fhi.no/))  Social- och hälsovårdsministeriet (<https://stm.fi/sv/framsida>)  THL (Finnish National Institute for Health and Welfare (<https://thl.fi/en/web/thlfi-en>)  Myndigheten för Vård och omsorgsanalys –(<https://www.vardanalys.se/>)  Inspektionen för vård och omsorg (<https://www.ivo.se>)  Post- och telestyrelsen (<https://pts.se>)  Datainspektionen (<https://www.datainspektionen.se>)  MSB (<https://www.msb.se>)  Vinnova (<https://www.vinnova.se>)  Myndigheten för delaktighet (<https://www.mfd.se/>)  E-hälsomyndigheten (<https://www.ehalsomyndigheten.se/>)  Folkhälsomyndigheten (<https://www.folkhalsomyndigheten.se>)  Jämställdhetsmyndigheten (<https://www.jamstalldhetsmyndigheten.se>)  Myndigheten för digital förvaltning (<https://www.digg.se>)  Swedish Standards Institute (<https://www.sis.se>)  Upphandlingsmyndigheten (<https://www.upphandlingsmyndigheten.se>)  Other research-based institutes and organisations:  Nordens välfärdscenter (<https://nordicwelfare.org/>)  SINTEF( <https://www.sintef.no/>)  RISE (<https://www.ri.se/sv>)  Nationalt Netveark for velfaerdsteknologi (<https://www.carenet.nu/>)  Welfare Tech (<https://en.welfaretech.dk/>)  Nasijonalt senter for e-helseforskning (<https://ehealthresearch.no>)  Other organisations and publication sites  Google Scholar (<https://scholar.google.com/>)  Posifon (<https://posifon.se/kunskapsbank/>) | 452  (109 duplicates removed) |
| 7. Snow-balling iteration 4 | Relevant references from larger grey literature search. | 4 (1 duplicate removed) |

**References (presented in the order as they occur in above, also found in the article text and the article full reference list).**

16. Carswell W, McCullagh PJ, Augusto JC, Martin S, Mulvenna MD, Zheng H, et al. A review of the role of assistive technology for people with dementia in the hours of darkness. Technol Health Care 2009;17(4):281-304. [doi: 10.3233/THC-2009-0553] [Medline: 19822946]

17. Hall A, Wilson CB, Stanmore E, Todd C. Implementing monitoring technologies in care homes for people with dementia: A qualitative exploration using Normalization Process Theory. Int J Nurs Stud 2017 Jul;72:60-70 [FREE Full text] [doi: 10.1016/j.ijnurstu.2017.04.008] [Medline: 28494333]

18. Landau R, Auslander GK, Werner S, Shoval N, Heinik J. Who should make the decision on the use of GPS for people with dementia? Aging Ment Health 2011 Jan;15(1):78-84. [doi: 10.1080/13607861003713166] [Medline: 20924823]

10. Røhne M, Tone D, Øystein D. GPS som varslings-og lokaliseringsteknologi i helse og omsorg. SINTEF. 2017. URL: <https://sintef.brage.unit.no/sintef-xmlui/handle/11250/2478904> [accessed 2021-06-15]

19. Larsson P, Gill M, Lind MS. Framtidens teknik i omsorgens tjänst. SOU. 2020. URL: <https://www.regeringen.se/494156/contentassets/576aa4588db340b0ad052537ae90511d/framtidens-teknik-i-omsorgens-tjanst-sou-2020_14.pdf> [accessed 2021-10-07]
