# Supplementary material for: Mobile Safety Alarms Based on GPS Technology in the Care of Older Adults: Systematic Review of Evidence Based on a General Evidence Framework for Digital Health Technologies
Source: J Med Internet Res. 2021 Oct 11;23(10):e27267. doi: 10.2196/27267 (PMC8546532; doi:10.2196/27267)
Supplement: Multimedia Appendix 2 [file jmir_v23i10e27267_app2.docx]

***Initial search in scientific databases***

**Academic Search Elite (EBSCOhost), 2020-08-20**

| # | Searches |
| --- | --- |
| 1 | TI ( aged OR elder* OR "older adult*" OR "older person*" OR ageing OR aging OR senior* ) OR AB ( aged OR elder* OR "older adult*" OR "older person*" OR ageing OR aging OR senior* ) OR ( DE "OLDER people" OR DE "FRAIL elderly" OR DE "AGING" ) |
| 2 | (TI alarm* OR AB alarm* OR DE "ALARMS") |
| 3 | TI ( geofencing OR "global positioning system*" OR "positioning technolog*" OR "localization system*" OR "localization technolog*" OR "localisation system*" OR "localisation technolog*" OR "location tracking system*" OR "location tracking device*" OR "location tracking technolog*" ) OR AB ( geofencing OR "global positioning system*" OR "positioning technolog*" OR "localization system*" OR "localization technolog*" OR "localisation system*" OR "localisation technolog*" OR "location tracking system*" OR "location tracking device*" OR "location tracking technolog*" ) OR DE "GLOBAL Positioning System") |
| 4 | 1 AND 2 AND 3 |
| 5 | Limit: Publication date from 2005-2020 |

**APA PsycInfo (EBSCOhost), 2020-08-20**

| # | Searches |
| --- | --- |
| 1 | TI ( aged OR elder* OR "older adult*" OR "older person*" OR ageing OR aging OR senior* ) OR AB ( aged OR elder* OR "older adult*" OR "older person*" OR ageing OR aging OR senior* ) OR ( (DE "Aging") OR (DE "Older Adulthood")) |
| 2 | (TI alarm* OR AB alarm*) |
| 3 | TI ( geofencing OR "global positioning system*" OR "positioning technolog*" OR "localization system*" OR "localization technolog*" OR "localisation system*" OR "localisation technolog*" OR "location tracking system*" OR "location tracking device*" OR "location tracking technolog*" ) OR AB ( geofencing OR "global positioning system*" OR "positioning technolog*" OR "localization system*" OR "localization technolog*" OR "localisation system*" OR "localisation technolog*" OR "location tracking system*" OR "location tracking device*" OR "location tracking technolog*" ) |
| 4 | 1 AND 2 AND 3 |
| 5 | Limit: Publication date from 2005-2020 |

**Applied Social Sciences Index & Abstracts –** **ASSIA (ProQuest), 2020-08-20**

| # | Searches |
| --- | --- |
| 1 | ti(aged OR elder* OR "older adult*" OR "older person*" OR ageing OR aging OR senior*) OR ab(aged OR elder* OR "older adult*" OR "older person*" OR ageing OR aging OR senior*) OR (MAINSUBJECT.EXACT("Elderly people") OR MAINSUBJECT.EXACT("Ageing")) |
| 2 | (ti(alarm*) OR ab(alarm*) OR MAINSUBJECT.EXACT("Alarms") |
| 3 | ti(geofencing OR "global positioning system*" OR "positioning technolog*" OR "localization system*" OR "localization technolog*" OR "localisation system*" OR "localisation technolog*" OR "location tracking system*" OR "location tracking device*" OR "location tracking technolog*") OR ab(geofencing OR "global positioning system*" OR "positioning technolog*" OR "localization system*" OR "localization technolog*" OR "localisation system*" OR "localisation technolog*" OR "location tracking system*" OR "location tracking device*" OR "location tracking technolog*") |
| 4 | 1 AND 2 AND 3 |
| 5 | Limit: Publication date from 2005-2020 |

**CINAHL Plus (EBSCOHost), 2020-08-20**

| # | Searches |
| --- | --- |
| 1 | TI ( aged OR elder* OR "older adult*" OR "older person*" OR ageing OR aging OR senior* ) OR AB ( aged OR elder* OR "older adult*" OR "older person*" OR ageing OR aging OR senior* ) OR ( (MH "Aged+") OR (MH "Aging") ) |
| 2 | TI alarm* OR AB alarm* OR ( (MH "Security Measures, Electronic") OR (MH "Emergency Medical Service Communication Systems") ) |
| 3 | TI ( geofencing OR "global positioning system*" OR "positioning technolog*" OR "localization system*" OR "localization technolog*" OR "localisation system*" OR "localisation technolog*" OR "location tracking system*" OR "location tracking device*" OR "location tracking technolog*" ) OR AB ( geofencing OR "global positioning system*" OR "positioning technolog*" OR "localization system*" OR "localization technolog*" OR "localisation system*" OR "localisation technolog*" OR "location tracking system*" OR "location tracking device*" OR "location tracking technolog*" ) OR (MH "Global Positioning System") |
| 4 | 1 AND 2 AND 3 |
| 5 | Limit: Publication date from 2005-2020 |

**Cochrane Library (www.cochranelibrary.com), 2020-08-20**

| # | Searches |
| --- | --- |
| 1 | aged OR elder* OR "older adult*" OR "older person*" OR ageing OR aging OR senior*):ti,ab,kw OR MeSH descriptor: [Aged] in all MeSH products OR MeSH descriptor: [Aged] in all MeSH products |
| 2 | (alarm*):ti,ab,kw |
| 3 | (geofencing OR "global positioning system*" OR "positioning technolog*" OR "localization system*" OR "localization technolog*" OR "localisation system*" OR "localisation technolog*" OR "location tracking system*" OR "location tracking device*" OR "location tracking technolog*"):ti,ab,kw OR MeSH descriptor: [Geographic Information Systems] this term only |
| 4 | 1 AND 2 AND 3 |
| 5 | Limit: Publication date from 2005-2020 |

**International Bibliography of the Social Sciences - IBSS (ProQuest), 2020-08-20**

| # | Searches |
| --- | --- |
| 1 | ti(aged OR elder* OR "older adult*" OR "older person*" OR ageing OR aging OR senior*) OR ab(aged OR elder* OR "older adult*" OR "older person*" OR ageing OR aging OR senior*) OR (MAINSUBJECT.EXACT("Older people") OR MAINSUBJECT.EXACT("Aging")) |
| 2 | ti(alarm*) OR ab(alarm*) OR MAINSUBJECT.EXACT("Alarms") |
| 3 | ti(geofencing OR "global positioning system*" OR "positioning technolog*" OR "localization system*" OR "localization technolog*" OR "localisation system*" OR "localisation technolog*" OR "location tracking system*" OR "location tracking device*" OR "location tracking technolog*") OR ab(geofencing OR "global positioning system*" OR "positioning technolog*" OR "localization system*" OR "localization technolog*" OR "localisation system*" OR "localisation technolog*" OR "location tracking system*" OR "location tracking device*" OR "location tracking technolog*") OR MAINSUBJECT.EXACT("Global positioning systems--GPS") |
| 4 | 1 AND 2 AND 3 |
| 5 | Limit: Publication date from 2005-2020 |

**IEEE (https://ieeexplore.ieee.org), 2020-08-20**

| # | Searches |
| --- | --- |
| 1 | ("All Metadata":aged OR elder* OR "older adult*" OR "older person*" OR ageing OR aging OR senior*) |
| 2 | ("All Metadata":alarm*) |
| 3 | ("All Metadata":geofencing OR "global positioning system" OR "positioning technology" OR "localization system" OR "localization technology" OR "localisation system" OR "localisation technology" OR "location tracking system" OR "location tracking device" OR "location tracking technology") |
| 4 | 1 AND 2 AND 3 |
| 5 | Limit: Publication date from 2005-2020 |

**PubMed (https://pubmed.ncbi.nlm.nih.gov), 2020-08-20**

| # | Searches |
| --- | --- |
| 1 | (aged[Title/Abstract] OR elder*[Title/Abstract] OR "older adult*"[Title/Abstract] OR "older person*"[Title/Abstract] OR ageing[Title/Abstract] OR aging[Title/Abstract] OR senior*[Title/Abstract]) OR ("Aged"[Mesh] OR "Aging"[Mesh]) |
| 2 | alarm*[Title/Abstract] |
| 3 | geofencing[Title/Abstract] OR "global positioning system*"[Title/Abstract] OR "positioning technolog*"[Title/Abstract] OR "localization system*"[Title/Abstract] OR "localization technolog*"[Title/Abstract] OR "localisation system*"[Title/Abstract] OR "localisation technolog*"[Title/Abstract] OR "location tracking system*"[Title/Abstract] OR location tracking device*[Title/Abstract] OR location tracking technolog*[Title/Abstract] OR "Geographic Information Systems"[Mesh] |
| 4 | 1 AND 2 AND 3 |
| 5 | Limit: Publication date from 2005-2020 |

**Scopus (www.scopus.com), 2020-08-20**

| # | Searches |
| --- | --- |
| 1 | TITLE-ABS-KEY ( aged  OR  elder*  OR  "older adult*"  OR  "older person*"  OR  ageing  OR  aging  OR  senior* ) |
| 2 | TITLE-ABS-KEY ( alarm* ) |
| 3 | TITLE-ABS-KEY ( geofencing  OR  "global positioning system*"  OR  "positioning technolog*"  OR  "localization system*"  OR  "localization technolog*"  OR  "localisation system*"  OR  "localisation technolog*"  OR  "location tracking system*"  OR  "location tracking device*"  OR  "location tracking technolog*" ) |
| 4 | 1 AND 2 AND 3 |
| 5 | Limit: Publication date from 2005-2020 |

**SocINDEX (EBSCOHost), 2020-08-20**

| # | Searches |
| --- | --- |
| 1 | TI ( aged OR elder* OR "older adult*" OR "older person*" OR ageing OR aging OR senior* ) OR AB ( aged OR elder* OR "older adult*" OR "older person*" OR ageing OR aging OR senior* ) OR ( (DE "OLDER people") OR (DE "AGING") ) |
| 2 | TI alarm* OR AB alarm* |
| 3 | TI ( geofencing OR "global positioning system*" OR "positioning technolog*" OR "localization system*" OR "localization technolog*" OR "localisation system*" OR "localisation technolog*" OR "location tracking system*" OR "location tracking device*" OR "location tracking technolog*" ) OR AB ( geofencing OR "global positioning system*" OR "positioning technolog*" OR "localization system*" OR "localization technolog*" OR "localisation system*" OR "localisation technolog*" OR "location tracking system*" OR "location tracking device*" OR "location tracking technolog*" ) |
| 4 | 1 AND 2 AND 3 |
| 5 | Limit: Publication date from 2005-2020 |

**Sociological Abstracts/ Social Services Abstracts (ProQuest), 2020-08-20**

| # | Searches |
| --- | --- |
| 1 | ti (aged OR elder* OR "older adult*" OR "older person*" OR ageing OR aging OR senior*) OR ab(aged OR elder* OR "older adult*" OR "older person*" OR ageing OR aging OR senior*) OR (MAINSUBJECT.EXACT("Elderly") OR MAINSUBJECT.EXACT("Aging")) |
| 2 | ti(alarm*) OR ab(alarm*) |
| 3 | ti(geofencing OR "global positioning system*" OR "positioning technolog*" OR "localization system*" OR "localization technolog*" OR "localisation system*" OR "localisation technolog*" OR "location tracking system*" OR "location tracking device*" OR "location tracking technolog*") OR ab(geofencing OR "global positioning system*" OR "positioning technolog*" OR "localization system*" OR "localization technolog*" OR "localisation system*" OR "localisation technolog*" OR "location tracking system*" OR "location tracking device*" OR "location tracking technolog*") OR MAINSUBJECT.EXACT("Geographic Information Systems") |
| 4 | 1 AND 2 AND 3 |
| 5 | Limit: Publication date from 2005-2020 |

**Web of Science Core Collection (www.webofknowledge.com), 2020-08-20**

| # | Searches |
| --- | --- |
| 1 | TOPIC: (aged OR elder* OR "older adult*" OR "older person*" OR ageing OR aging OR senior*) |
| 2 | TOPIC: (alarm*) |
| 3 | TOPIC: (geofencing OR "global positioning system*" OR "positioning technolog*" OR "localization system*" OR "localization technolog*" OR "localisation system*" OR "localisation technolog*" OR "location tracking system*" OR "location tracking device*" OR "location tracking technolog*") |
| 4 | 1 AND 2 AND 3 |
| 5 | Limit: Publication date from 2005-2020 |

***Initial search in grey literature databases***

**Base (https://www.base-search.net), 2020-09-08**

| # | Searches |
| --- | --- |
| 1 | (aged OR elder* OR old* OR ageing OR aging OR senior*) |
| 2 | (Alarm*) |
| 3 | (geofencing OR "global positioning system*" OR "positioning technolog*" OR "localization system*" OR "localization technolog*" OR "localisation system*" OR "localisation technolog*" OR "location tracking") |
| 4 | 1 AND 2 AND 3 |

**Google Scholar (https://scholar.google.com), 2020-09-08**

| # | Searches |
| --- | --- |
| 1 | elder alarm "global positioning system" |
| 3 | Limit: Publication date from 2005-2020 |
| 5 | Limit: First 50 records downloaded |

**Open Grey (http://www.opengrey.eu), 2020-09-08**

| # | Searches |
| --- | --- |
| 1 | (aged OR elder* OR "older adult*" OR "older person*" OR ageing OR aging OR senior*) |
| 2 | (geofencing OR "global positioning system*" OR "positioning technolog*" OR "localization system*" OR "localization technolog*" OR "localisation system*" OR "localisation technolog*" OR "location tracking system*" OR "location tracking device*" OR "location tracking technolog*") |
| 3 | 1 AND 2 |

**OAIster (http://oaister.worldcat.org), 2020-09-09**

| # | Searches |
| --- | --- |
| 1 | kw:(elder* OR "older adult*" OR "older person*") |
| 2 | kw:(geofencing OR "global positioning system*" OR "positioning technolog*" OR "localization system*" OR "localization technolog*" OR "localisation system*" OR "localisation technolog*" OR "location tracking system*" OR "location tracking device*" OR "location tracking technolog*") |
| 3 | 1 AND 2 |
| 4 | Limit: Publication date from 2005-2020 |

**DART-Europé (www.dart-europe.eu), 2020-09-09**

| # | Searches |
| --- | --- |
| 1 | (aged OR elder* OR "older adult*" OR "older person*" OR ageing OR aging OR senior*) |
| 2 | (Alarm*) AND (geofencing OR "global positioning system*" OR "positioning technolog*" OR "localization system*" OR "localization technolog*" OR "localisation system*" OR "localisation technolog*" OR "location tracking system*" OR "location tracking device*" OR "location tracking technolog*") |
| 3 | 1 AND 2 |

**ProQuest Dissertations & Theses Global (ProQuest), 2020-09-09**

| # | Searches |
| --- | --- |
| 1 | noft(aged OR elder* OR "older adult*" OR "older person*" OR ageing OR aging OR senior*) |
| 2 | noft(alarm*) |
| 3 | noft(geofencing OR "global positioning system*" OR "positioning technolog*" OR "localization system*" OR "localization technolog*" OR "localisation system*" OR "localisation technolog*" OR "location tracking system*" OR "location tracking device*" OR "location tracking technolog*") |
| 4 | 1 AND 2 AND 3 |

**WHO ICTRP (https://apps.who.int/trialsearch), 2020-09-09**

| # | Searches |
| --- | --- |
| 1 | This page is temporarly unavailable or there has been an error. We are sorry for this, please try again later or try changing your search criteria. |

**ClinicalTrials.gov (www.clinicaltrials.gov), 2020-09-09**

| # | Searches |
| --- | --- |
| 1 | (elder* OR older OR aged) |
| 2 | (geofencing OR "global positioning system*" OR "positioning technolog*" OR "localization system*" OR "localization technolog*" OR "localisation system*" OR "localisation technolog*" OR "location tracking") |
| 3 | 1 AND 2 |

**INTERNATIONAL HTA DATABASE (www.inahta.org/hta-database), 2020-09-09**

| # | Searches |
| --- | --- |
| 1 | geofencing OR "global positioning system*" OR "positioning technolog*" OR "localization system*" OR "localization technolog*" OR "localisation system*" OR "localisation technolog*" OR "location tracking" |
