# Supplementary material for: Mobile Safety Alarms Based on GPS Technology in the Care of Older Adults: Systematic Review of Evidence Based on a General Evidence Framework for Digital Health Technologies
Source: J Med Internet Res. 2021 Oct 11;23(10):e27267. doi: 10.2196/27267 (PMC8546532; doi:10.2196/27267)
Supplement: Multimedia Appendix 3 [file jmir_v23i10e27267_app3.docx]

|  | Minimum evidence- and best practice standard of evidence category^a^ | Findings | Studies, n (%) |
| --- | --- | --- | --- |
|  |  |  |  |
| **Tier 1: Credibility with health, social care professionals** |  |  |  |
|  | *Minimum evidence standard:* Be able to show that the DHT has a plausible mode of action that is viewed as useful and relevant by professional experts or expert groups in the relevant field. Either: show that relevant clinical or social care professionals working within the national health and social care system have been involved in the design, development or testing of the DHT, or show that relevant clinical or social care professionals working within the national health and social care system have been involved in signing-off the DHT, indicating their informed approval of the DHT.  *Best practice standard:* Published or publicly available evidence documenting the role of relevant national health or social care experts in the design, development, testing or sign-off of the DHT. | - Minimum evidence standards show that relevant social care professionals were involved in the design, development or testing of the GPS devices - In 12 (75%) [22,24,25,28-36], of the included studies, social care professionals were involved in the testing of the GPS devices to varied extent. | 12 (75) |
| **Tier 1: Relevance to current pathways in health/social care system** |  |  |  |
|  | *Minimum evidence standard:* Evidence to show that the DHT has been successfully piloted in the national health and social care system, showing that it is relevant to current care pathways and service provision in the national. Also, evidence that the DHT is able to perform its intended function to the scale needed (for example, having servers that can scale to manage the expected number of users).  *Best practice standard:* Evidence to show successful implementation of the DHT in the national health and social care system. | - Minimum and best practice evidence standards show that GPS devices have been successfully piloted or implemented in social care systems. This was described in 10 (63%) of the included studies. Of these ten, three were performed in Sweden [22,32,33], six in Norway [28-31,34,35], and one in Denmark [36]. All of them were part of larger projects supporting development of products, services, and decision-making processes to support OAs^b^and their families in their homes. Most of those projects were part of national government programs that aimed to stimulate the use of welfare technology. | 10 (63) |
| **Tier 1: Acceptability with users** |  |  |  |
|  | *Minimum evidence standard:* Be able to show that representatives from intended user groups were involved in the design, development or testing of the DHT. Provide data to show user satisfaction with the DHT.  *Best practice standard:* Published or publically available evidence to show that representatives from intended user groups were involved in the design, development or testing of the DHT and to show that users are satisfied with the DHT | - Best practice evidence shows that representatives from the intended user groups (persons with dementia and OAs) were involved in the design, development, or testing of the DHT^c^ and to show that users are satisfied with the DHT. - Representatives from the intended user groups (OAs in general or persons with dementia) were involved in testing of the GPS alarms in 15 (94%) of the studies (ie, all the included studies except [29]). - Six (38%) of these studies showed that the users were satisfied with the alarms: 77% of the CGs^d^ of persons with dementia stated that they would recommend the use of GPS alarms in in the Pot et al study [26]; 97% of the OAs who participated in the Røhne at al study [28] and 90% of the OAs in the Røhne at al study [35] stated that they were satisfied with the alarm. - All older users in the Ausen et al study [30] would recommend others in similar situations to use the GPS alarm. - User satisfaction was confirmed in the interviews in the Milne et al study [24] and in the values identified in the Boysen et al study [31]. | 6 (38) |
| **Tier 1: Equalities considerations** |  |  |  |
|  | *Minimum evidence standard:* Evidence, if relevant, that the DHT: Contributes to challenging health inequalities in the UK health and social care system, or improving access to care among hard-to-reach populations; Contribute to promoting equality, eliminating unlawful discrimination and fostering good relations between people with protected characteristics (as described in the 2010 Equalities Act) and others.  *Best practice standard:* Show evidence of the DHT being used in hard-to-reach populations | - *No information retrieved from included studies. Socioeconomic aspects not addressed.* - However, persons with dementia and OAs may be considered vulnerable groups. | 0 |
| **Tier 1: Accurate and reliable measurements (if relevant)** |  |  |  |
|  | *Minimum evidence standard:* Data or analysis which shows that the data generated or recorded by the DHT is: accurate; reproducible; relevant to the range of values expected in the target population. Also, data showing that the DHT is able to detect clinically relevant changes or responses.  *Best practice standard:* As for the minimum evidence standard | - *No information retrieved from included studies. Technical validations not included in the review. All included studies used commercial products.* | 0 |
| **Tier 1: Accurate and reliable transmission of data (if relevant)** |  |  |  |
|  | *Minimum evidence standard:* Data or analysis which shows that the data generated or recorded by the DHT is: accurate; reproducible; relevant to the range of values expected in the target population. Also, data showing that the DHT is able to detect clinically relevant changes or responses.  *Best practice standard*: As for the minimum evidence standard | - *No information retrieved from included studies. Technical validations not included in the review. All included studies used commercial products.* | 0 |
| **Tier 2: Reliable information content** |  |  |  |
|  | *Minimum evidence standard:* Be able to show that any health information provided by the DHT is: valid (aligned to best available sources, such as NICE guidance, relevant professional organizations or recognized UK patient organizations, and appropriate for the target population); accurate; up to date; reviewed and updated by relevant experts at defined intervals, such as every year; sufficiently comprehensive.  *Best practice standard:* Evidence of endorsement, accreditation or recommendation by NICE, NHS England, a relevant professional body or recognized UK patient organization. Alternatively, evidence that the information content has been validated though an independent accreditation such as The Information Standard or HONcode certification. | - *Minimum and best practice standards category is not relevant for GPS devices because they do not provide general information or advice to users concerning health, healthy living, lifestyle, diseases, illnesses, or conditions.* - For reliability of information on user position and emergency situations, data on user testing were provided by one study (6%) [28], and data on CGs’ perceptions of the accuracy of the GPS information were provided by two (13%) studies. For example, relatives and staff in the Øderud et al study [34] had experienced slow or unreliable information on the user’s position. Moreover, [30] reported cases of poor mobile coverage that had resulted in failures in updating user position. | 0 |
| **Tier 2: Ongoing data collection to show usage** |  |  |  |
|  | *Minimum evidence standard:* Commitment to ongoing data collection to show **usage** of the DHT in the target population, and commitment to share, when available, with relevant decision-makers such as commissioners in a clear and useful format.  *Best practice standard:* Evidence that data on **usage** is being collected in line with the minimum standards and can be made available to relevant decision-makers. | - *Cannot be assessed from the included studies.* - Evidence of ongoing data collection (required according to evidence standards for the category) was not reported in the included studies. However, 10 (63%) of the included studies presented data on use on specific occasions related to the interventions. - In all, three studies (19%) reported quantitative data on usage period: [30] and [34] presented the number of participants who had used GPS trackers for up to 1 year and between 1 and 2 years, respectively. [33] reported the number of days that each participant had used the GPS trackers (mean 158 days, median 210 days, and range 37-260 days). - A total of three (19%) studies [22,25,28] included system logs in the collection of data to investigate use. Interestingly, [22] saw that the extent to which persons with dementia used mobile phone–based GPS varied widely among the participants. Moreover, [28] described that logs from the technical systems have been thoroughly analyzed to understand the role and function of users, alarm units, response center, CGs and relatives. - In all, four (25%) studies [23,24,26,27] based the data collection of use on the recall of the users or their CGs, and two of these [26,27] reported that the persons with dementia did not always take along the GPS devices (mobile phone or tracker worn on the belt) when going out and that the devices were not always switched on. | 0 |
| **Tier 2: Ongoing data collection to show value** |  |  |  |
|  | *Minimum evidence standard:*  Commitment to ongoing data collection to show user outcomes (if relevant) or user satisfaction (using non-patient identifiable information) to show ongoing value, and commitment to share, when available, with relevant decision-makers such as commissioners in a clear and useful format.  *Best practice standard:* Evidence that data on outcomes or user satisfaction is being collected in line with the minimum standard and can be made available to relevant decision-makers. | - *Cannot be assessed from the included studies.* - Evidence of ongoing data collection to show value (required according to evidence standards for the category) was not reported. However, 11 (69%) of the included studies presented data on use for values related to the health and welfare outcomes of users (OAs, persons with dementia, and CGs of persons with dementia) on specific occasions related to the interventions; one (6%) study [22] identified that CGs experienced that the persons with dementia had become more independent in outdoor activity; one (6%) study [24] identified that CGs and staff saw that GPS trackers could give persons with dementia in milder stages of dementia and their CGs increased freedom and decreased stress and anxiety; and one (6%) study [26] identified values perceived by some of the CRs^e^, including increased freedom and decreased worries and fewer conflicts with CGs when going outside alone. Moreover, the CGs experienced that they gave more freedom to the CR and some experienced fewer conflicts with the CR. - Another study (6%) [28] identified that more than 50% of the users thought that the GPS alarm helped to increase their freedom. - One study (6%) [29] noted that more than 50% of the participating staff perceived that GPS trackers for persons with dementia could, to some degree, free up time for service providers by reducing the number of inspections they carried out to see if the person is well, driving to and from the user and following the user on walks; [30] identified that all persons with dementia thought that GPS trackers enabled them to increase or maintain physical activity, to increase freedom in outdoor activities, and that all relatives experienced that the GPS trackers increased their feelings of safety when leaving the person with dementia by themselves; and [31] identified positive values of GPS trackers both in shared housing for persons with dementia, including freedom for persons with dementia, decreased stress and anxiety for employees, time savings for staff and cost reduction, and for home users, including increased security, with, in some cases, increased outdoor activity and CG relief. - Another study [32] identified that 5 of the 8 GPS tracker users experienced increased security and could continue to live at home for a longer periods. In addition, 5 of the 8 relatives experienced fewer concerns and worries; [34] noted that most of the users perceived that the GPS trackers provide security (for user, GG and staff), increase freedom for the user and sometimes also the CG, as well as help the user to be physically active and maintain activity level; [35] identified that the majority of the GPS alarm users experienced that it increased their safety and freedom in daily life; and [36] identified that the GPS trackers increased security and quality of life for persons with dementia and their CGs. | 0 |
| **Tier 2: Quality and safeguarding** |  |  |  |
|  | *Minimum evidence standard:* Show that appropriate safeguarding measures are in place around peer-support and other communication functions within the platform. Describe who has access to the platform and their roles within the platform. Describe why these people or groups are suitable and qualified to have access. Describe any measures in place to ensure safety in peer-to-peer communication, for example through user agreements or moderation.  *Best practice standard:* As for the minimum evidence standard. | - *Cannot be assessed from the included studies.* - However, three (19%) of the included studies had a study aim or presented data related to system-level quality and safeguarding: [30] and [34] presented the service model for implementation of GPS trackers in the homes of older adults, which included safeguarding measures taken by the municipality. Moreover, [34] presented data on the roles of different actors (users, relatives, and alarm centers) in charging and administration of the alarm as well as locating and retrieving the user, if necessary. - One study (6%) [28] described the establishment of an initial test routine to encourage users to regularly trigger the alarm when out walking. | 0 |
| **Tier 3a: Demonstrating effectiveness: outcomes/improvements in outcomes** |  |  |  |
|  | *Minimum evidence standard:*  High quality observational or quasi-experimental studies demonstrating relevant outcomes. These studies should present comparative data. Comparisons could include: relevant outcomes in a control group, use of historical controls, routinely collected data. Relevant outcomes may include: behavioral or condition-related user outcomes such as reduction in smoking or improvement in condition management; evidence of positive behavior change; user satisfaction.  *Best practice standard:*  High quality intervention study (quasi-experimental or experimental design) which incorporates a comparison group, showing improvements in relevant outcomes, such as: patient-reported outcomes (preferably using validated tools) including symptom severity or quality of life; other clinical measures of disease severity or disability; healthy behaviors; physiological measures; user satisfaction and engagement; health and social care resource use, such as admissions or appointments. The comparator should be a care option that is reflective of standard care in the current care pathway, such as a commonly used active intervention. | - Effectiveness is not demonstrated in outcomes or improvements in outcomes according to best practice standards: no increase in the frequency of OAs going outside; no signiﬁcant differences in changes in fear of falling, feelings of unsafety, or quality of life [27]. - Effectiveness is *not* demonstrated in outcomes/ improvements in outcomes according to minimum evidence standards. Indications were identified for the following:   - Decrease in time searching for person with dementia (from a mean of 3-4 hours per event to 40 minutes) [24].   - Increase in the number of days that person with dementia was engaged in independent outdoor activity (three cases, no statistics available) [25].   - Decrease in role-overload of CGs of persons with dementia (*P*=.126; d=–0.25 for all CGs, and *P*=.119; d=–0.34 for CGs who could reach CR with the mobile alarm) and in feelings of worry (*P*=.08; d=–0.32 for all CGs, and *P*=.057; d=–0.46 for CGs who could reach CR with the alarm) [26].   - Reduction in costs for care of persons with dementia because of prolonged time that the person could live independently instead of in special housing (up to 3 months) [32,33,36].   - Difference in mean CG burden between relatives of persons with dementia using and not using GPS (*P*=.04) was indicated in small samples because a crossover design was used [37]. - Outcomes investigated with negative results:   - Activity of person with dementia: reduced because of disease progression [22].   - Burden and quality of life for CG of person with dementia: no significant changes [23]. | - 1 (6) - 7 (44) - 2 (12) |
| **Tier 3a: Use of appropriate behavior change techniques (if relevant)** |  |  |  |
|  | *Minimum evidence standard:* Be able to show that the techniques used in the DHT are: consistent with recognized behavior change theory and recommended practice (aligned to guidance from NICE or relevant professional organizations) appropriate for the target population.  *Best practice standard:* Published qualitative or quantitative evidence showing that the techniques used in the DHT are: based on published and recognized effective behavior change techniques aligned with recommended practice appropriate for the target population. | - *Not relevant for GPS devices: no behavior change techniques used* | 0 |
| **Tier 3b: Demonstrating effectiveness: improvements in outcomes** |  |  |  |
|  | *Minimum evidence standard:* High quality intervention study (experimental or quasi- experimental design) showing improvements in relevant outcomes, such as: diagnostic accuracy; patient-reported outcomes (preferably using validated tools) including symptom severity or quality of life; other clinical measures of disease severity or disability; healthy behaviors; physiological measures; user satisfaction and engagement. Generic outcome measures may also be useful when reported alongside condition-specific outcomes. The comparator should be a care option that is reflective of the current care pathway, such as a commonly used active intervention.  *Best practice standard:* High quality randomized controlled study or studies done in a setting relevant to the national health and social care system, comparing the DHT with a relevant comparator and demonstrating consistent benefit including in clinical outcomes in the target population, using validated condition-specific outcome measures. Alternatively, a well-conducted meta-analysis of randomized controlled studies if there are enough available studies on the DHT. | - Effectiveness is *not* demonstrated in improvements in outcomes according to minimum evidence or best practice standards (see above) | 0 |

^a^the word “UK” has here has been replaced by “national”

^b^OA: older adult.

^c^DHT: digital health technology.

^d^CG: caregiver.

^e^CR: care receiver

**References (presented in the order as they occur in above, also found in the article text and the article full reference list)**

22. Magnusson L, Sandman L, Gustav RK, Hanson E. Extended safety and support systems for people with dementia living at home. J Assist Technol 2014 Dec 9;8(4):188-206.

23. Megges H, Freiesleben SD, R.sch C, Knoll N, Wessel L, Peters O. User experience and clinical effectiveness with two wearable global positioning system devices in home dementia care. Alzheimers Dement (N Y) 2018 Nov 9;4:636-644

24. Milne H, van der Pol M, McCloughan L, Hanley J, Mead G, Starr J, et al. The use of global positional satellite location in dementia: a feasibility study for a randomised controlled trial. BMC Psychiatry 2014 May 30;14:160

25. Olsson A, Engstr.m M, .senl.f P, Skovdahl K, Lampic C. Effects of tracking technology on daily life of persons with dementia: three experimental single-case studies. Am J Alzheimers Dis Other Demen 2015 Feb;30(1):29-40

26. Pot AM, Willemse BM, Horjus S. A pilot study on the use of tracking technology: feasibility, acceptability, and benefits for people in early stages of dementia and their informal caregivers. Aging Ment Health 2012;16(1):127-134.

27. Scheffer AC, Scholte op Reimer WJ, van Dijk N, van Munster BC, Abu-Hanna A, Levi M, et al. Effect of a mobile safety alarm on going outside, feeling safe, fear of falling, and quality of life in community-living older persons: a randomized controlled trial. J Am Geriatr Soc 2012 May;60(5):987-989.

28. Røhne M, Boysen ES, Ausen D. Wearable and mobile technology for safe and active living. Stud Health Technol Inform 2017;237:133-139.

29. Sørli A. Effektstudie ved bruk av GPS til personer med demens. NTNU Open. 2013. URL: <https://ntnuopen.ntnu.no/ntnu-xmlui/handle/11250/143676> [accessed 2021-06-15]

30. Ausen D, Svag.rd I, Øderud T, Sørensen E, Stanarevic M. Selvstendig, trygg og aktiv i Larvik. Erfaringer fra

velferdsteknologiprosjektet i Larvik kommune. SINTEF. 2016. URL: <https://sintef.brage.unit.no/sintef-xmlui/handle/11250/2421428> [accessed 2021-06-15]

31. Boysen E, Støle N. Erfaringer og funn fra pilotering av velferdsteknologi i Stavanger kommune. SINTEF. 2016. URL: <https://sintef.brage.unit.no/sintef-xmlui/handle/11250/2421431> [accessed 2021-06-15]

32. Dahlberg .Nyttokostnadsanalys vid inf.rande av v.lf.rdsteknologi -exemplet Posifon. Hj.lpmedelsinstitutet. 2013. URL: <https://posifon.se/wp-content/uploads/2016/12/Nyttokostnadsanalys-inforande-valfardsteknologi.pdf> [accessed 2021-06-15]

33. Malmquist C. Utv.rdering av projekt F.rskrivning av larm i .stersund-ekonomi och proces. .stersund: payoff AB. 2013.URL: <https://docplayer.se/3797329-Utvardering-av-projekt-forskrivning-av-larm-i-ostersund-ekonomi-och-process-ostersund-2013-01-03.html> [accessed 2021-06-15]

34. Øderud T, Ausen D, Aketun S, Thorgersen M. GPS for trygghet, frihet og mestring. Fra prosjekt til drift-Bruk av GPS for lokalisering av personer med demens/kognitiv svikt. Sintef Rapport. 2017. URL: <https://sintef.brage.unit.no/sintef-xmlui/handle/11250/2489904> [accessed 2021-06-15]

35. Røhne M, Svag.rd I, Ausen D, Fossberg AB, Husebø I, Øverli T. Bo lenger hjemme med mobil trygghetsalarm? In: Erfaringer med mobil trygghetsalarm i Bærum kommune. Norway: SINTEF; 2015.

36. Demonstrationsprojekt med brug af GPS system i eget hjem. 2011. URL: <https://docplayer.dk/10583158-Demonstrationsprojekt-med-brug-af-gps-system-i-eget-hjem.html> [accessed 2021-06-15]

37. Ribas Miquel G, P.rez Gonz.lez A, Beltran Vilella M, Boix Roqueta E, Ferr. Munt. M, Reig Garcia G, et al. Uso de localizadores para personas mayores. Ariadna 2013;1(1):68-72. doi: 10.6035/ariadna.2013.1.11.
